# Vestibular Morphological Asymmetry Associated With Motion Sickness Susceptibility

**DOI:** 10.3389/fnins.2021.763040

**Published:** 2021-11-04

**Authors:** Takumi Harada, Tomoko Sugawara, Taeko Ito, Yoshiro Wada, Masaki Fukunaga, Norihiro Sadato, Stephen K. Larroque, Athena Demertzi, Steven Laureys, Hiroyuki Sakai

**Affiliations:** ^1^Toyota Central R&D Labs, Inc., Tokyo, Japan; ^2^Department of Otolaryngology-Head and Neck Surgery, Nara Medical University, Kashihara, Japan; ^3^Division of Cerebral Integration, Department of System Neuroscience, National Institute for Physiological Sciences, Okazaki, Japan; ^4^Coma Science Group, GIGA-Consciousness, GIGA Institute, University of Liège, Liege, Belgium; ^5^Physiology of Cognition Research Lab, GIGA-Consciousness, GIGA Institute, University of Liège, Liege, Belgium

**Keywords:** motion sickness, vestibular, morphology, asymmetry, inner ear, resting state, MRI

## Abstract

Sensory conflicts leading to motion sickness can occur not only between but also within sensory modalities. The vestibular organs are located in both left and right inner ears, and their misalignment can be a source of self-motion related sensory conflicts. In the current study, using inner ear magnetic resonance imaging, we examined whether morphological asymmetry of the bilateral vestibular organs was associated with motion sickness susceptibility. The results showed a larger position asymmetry of bilateral vestibular organs in individuals with high rather than low susceptibility. In addition, vestibular position asymmetry was associated with reciprocal interaction (negative resting state functional connectivity) between vestibular and visuocortical regions in lowly, but not highly, susceptible individuals. In conclusion, these findings suggest that vestibular morphological asymmetry can be a source of sensory conflicts in individuals with dysfunctional reciprocal visuo-vestibular interactions, a putative neural mechanism for resolving sensory conflicts.

## 1. Introduction

Motion sickness has afflicted humans throughout history and is predicted to occur more frequently in the era of self-driving cars (Diels and Bos, 2016). The unpleasant symptoms resulted from motion sickness can undermine the value of travel and consequently reduce the quality of everyday life (Henriques et al., 2014). The identification of individuals prone to motion sickness is important for taking proactive measures, such as recommending lower acceleration transportation or prescribing motion sickness medicine.

The sensory conflict theory (Oman, 1990; Bertolini and Straumann, 2016), the most widely accepted hypothesis for the pathogenesis of motion sickness, posits that motion sickness is caused by conflicted self-motion signals among different sensory modalities (e.g., vision and vestibular sense). However, the sensory conflicts causing motion sickness can occur within, as well as between, sensory modalities. Specifically, the vestibular organs are located in both the left and right inner ears, and their functional asymmetry can lead to sensory conflicts with respect to self-motion. For example, astronauts with higher otolith functional asymmetry are prone to motion sickness in space (Lackner and Dizio, 2006). Similarly, in terrestrial environments, bilateral asymmetry in vestibular sensitivity was associated with motion sickness susceptibility (Nooij et al., 2011; Sugawara et al., 2021).

Morphological asymmetry of the bilateral vestibular organs can also be a source of sensory conflicts leading to motion sickness. Using magnetic resonance imaging (MRI) for the inner ear, Hitier et al. Hitier et al. (2015) found that patients with idiopathic scoliosis, a disease presenting with spine deformity, showed greater morphological asymmetry in bilateral horizontal semicircular canal (SC) than healthy controls. Carry et al. (2020) recently replicated this result. Further, idiopathic scoliosis patients are also reported to be highly susceptible to motion sickness (Catanzariti et al., 2016). These lines of evidence suggest the association between vestibular morphological asymmetry and motion sickness susceptibility. However, no empirical research has tested this hypothesis so far.

Thus, the current study aimed at examining the impacts of morphological asymmetry of the bilateral vestibular organs on motion sickness susceptibility. First, we acquired high-resolution structural MRI images covering the bilateral inner ears from highly susceptible (HS) individuals to motion sickness and age/sex-matched low susceptibility (LS) controls. Then, we identified the left and right vestibular organs in each image and evaluated vestibular morphological asymmetry in various manners. Finally, we compared the vestibular morphological asymmetry indices between the two groups. In addition, using resting-state functional MRI, we also examined the impacts of the morphological asymmetry associated with motion sickness susceptibility on cortical vestibular networks.

## 2. Materials and Methods

### 2.1. Participants

A total of 72 healthy volunteers participated in this study. An online survey was used to recruit two age/sex-matched groups of participants who differed in motion sickness susceptibility, as assessed by the Motion Sickness Susceptibility Questionnaire (MSSQ) (Golding, 1998): the LS group (*n* = 36, 23 females and 13 males, aged 27 ± 8.9 years, MSSQ score 0.93 ± 1.9, mean ± standard deviation) and the HS group (*n* = 36, 23 females and 13 males, aged 27 ± 9.5 years, MSSQ score 107 ± 29). The thresholds of MSSQ scores for the LS and HS groups were determined by the first and fourth quantiles, respectively, of a healthy population (Golding, 1998). Participants had no history of neurological or mental disorders, and were right-handed as assessed by the Edinburgh Handedness Inventory (Oldfield, 1971). They were also diagnosed as having no clinical vestibular dysfunction based on a structured interview, subjective visual vertical test (tilt angle < |2.5°|) (Tribukait, 2006), and horizontal video head impulse test (gain > 0.7) (Yip et al., 2016), all of which were conducted by otolaryngologists (T.I. and Y.W.). All participants gave written informed consent before the experiments, and the study was approved by the ethics committees of both Toyota Central R&D Labs., Inc. and the National Institute for Physiological Sciences.

### 2.2. MRI Data Acquisition

For each participant, the structural image of vestibular organs was acquired using a 3-T MRI scanner (Verio, Siemens Medical System Inc., Erlangen, Germany) with a constructive interference in steady-state (CISS) sequence (Casselman et al., 1993) [repetition time (TR) = 6.79 ms, echo time (TE) = 3.03 ms, field of view (FOV) = 128 × 128 mm^2^, flip angle (FA) = g50°, 96 transverse slices, voxel size = 0.4 × 0.4 × 0.4 mm^3^, total acquisition time = 8.3 min]. To determine eye positions, a T1-weighted image covering the entire head was acquired successively using a magnetization-prepared rapid gradient-echo sequence with generalized autocalibrating partially parallel acquisitions (Griswold et al., 2002) (TR = 2,400 ms, TE = 1.78 ms, FOV = 240 × 256 mm^2^, FA = 8°, 240 sagittal slices, voxel size = 0.8 × 0.8 × 0.8 mm^3^, acceleration factor = 2, total acquisition time = 6.6 min).

Resting-state functional MRI data were also acquired using a multiband echo planar imaging (EPI) sequence (TR = 750 ms, TE = 31 ms, FOV = 189 × 189 mm^2^, FA = 55°, 72 contiguous transverse slices, voxel size = 2.1 × 2.1 × 2.1 mm^3^, and acceleration factor = 8). A total of 640 EPI images were collected, resulting in a scanning time of approximately 8.2 min. During scanning, participants were instructed to stay awake and lie still while keeping their eyes on a fixation point (a black Maltese cross on a gray background) without thinking of anything in particular. After scanning, spin-echo EPI images were acquired in both anterior-to-posterior and posterior-to-anterior phase-encoding directions, for spatial distortion correction of the EPI images (TR = 7,700 ms, TE = 60 ms, FOV = 189 × 189 mm^2^, FA = 78°, and voxel size = 2.1 × 2.1 × 2.1 mm^3^).

### 2.3. Morphological Asymmetry Analysis

**Figure 1:**
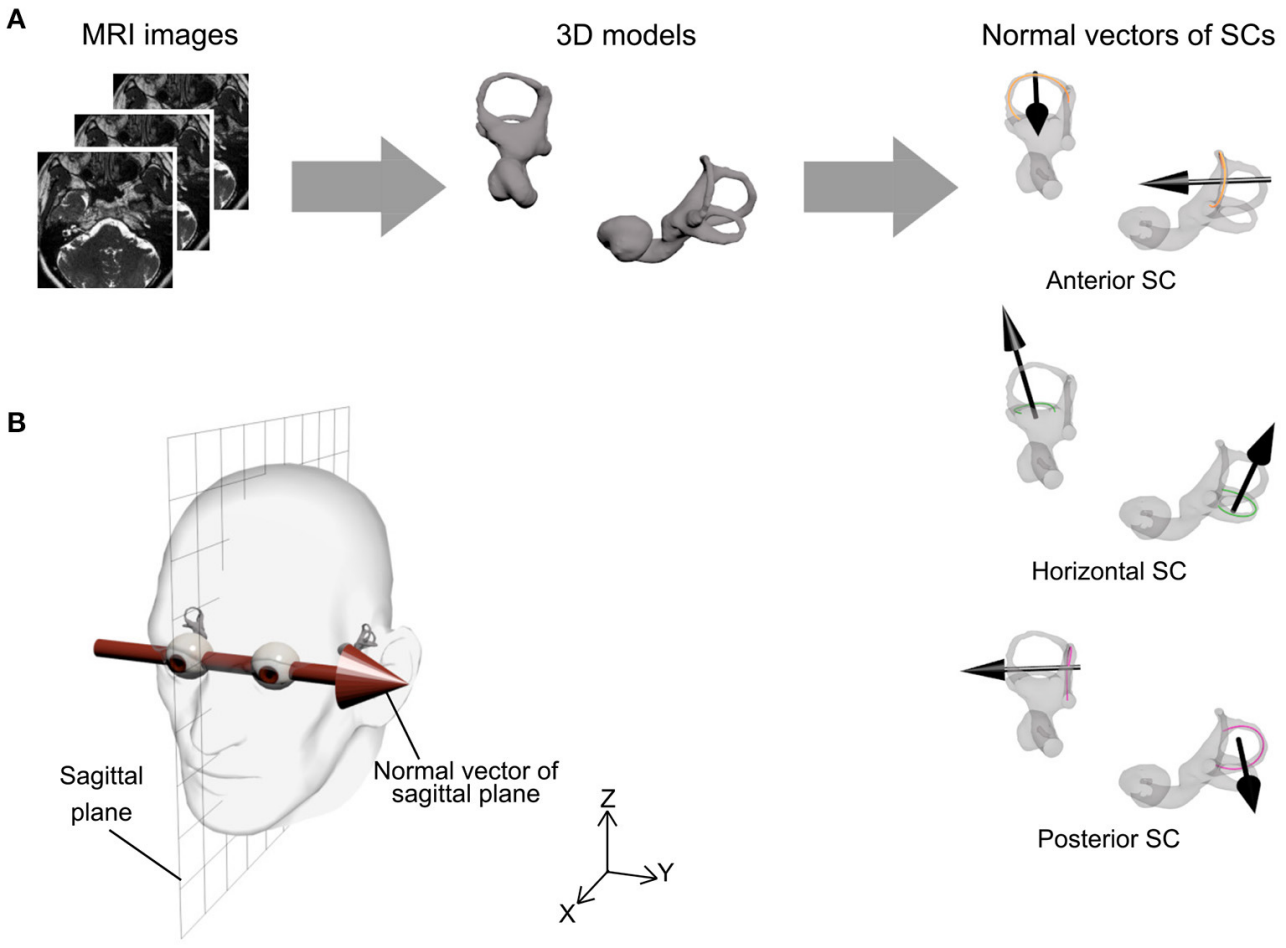
Procedure for semicircular canal (SC) orientation and sagittal plane. **(A)** Bilateral vestibular organ information was extracted from magnetic resonance imaging (MRI) scans and reconstructed as a three-dimensional model. For each SC, a centerline of SC was estimated by three-dimensional image processing software and the normal vector of the SC was then determined by principal components analysis (black arrow). **(B)** The sagittal plane was defined by a connecting vector of the eyes' centroids as normal vector (Y-axis) and the center of eyes' centroids as position (XZ plane).

**Figure 2:**
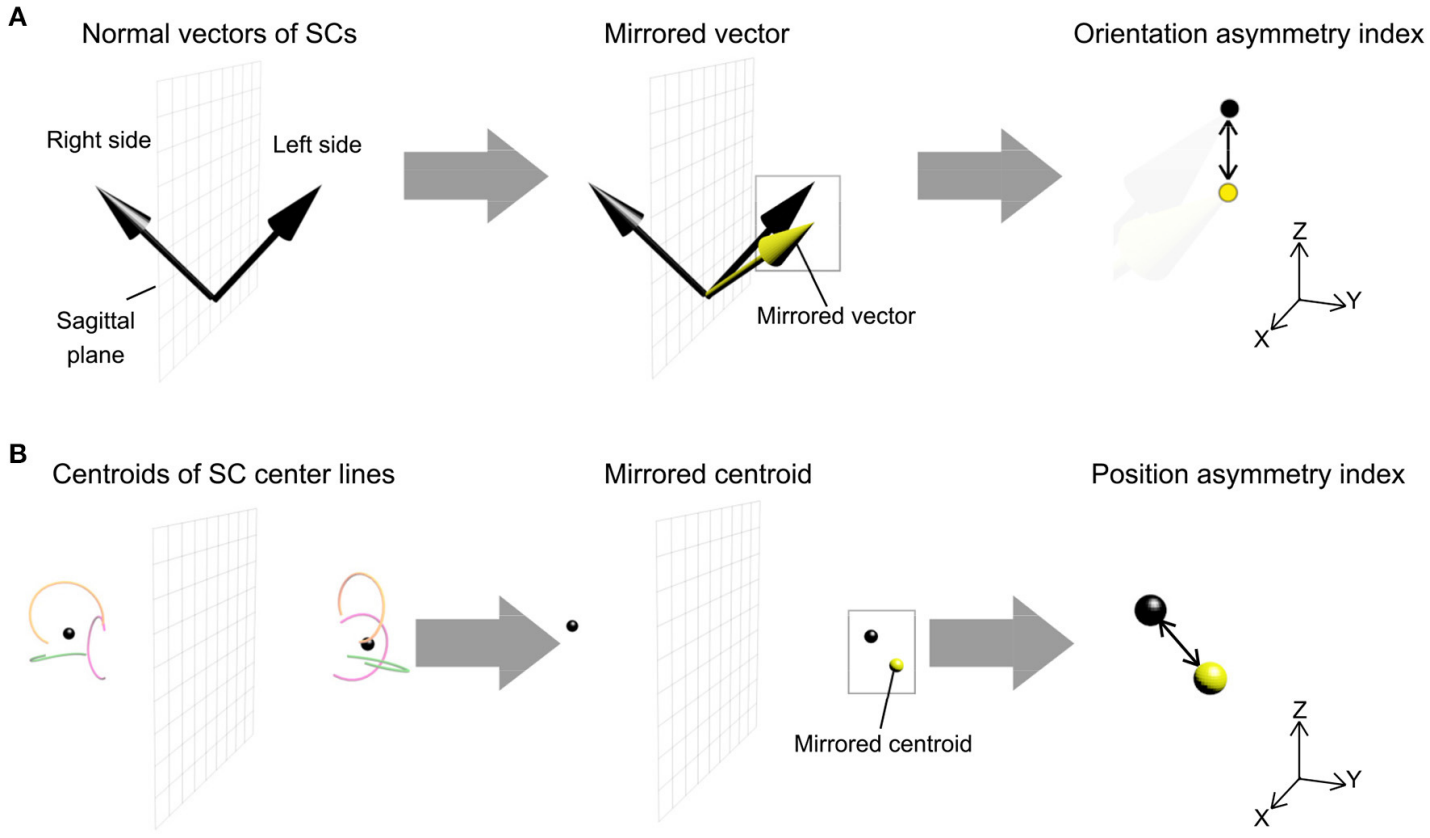
Procedure for measurement of morphological asymmetry in the bilateral vestibular organs. The morphological asymmetry was evaluated using the orientation asymmetry index and the position asymmetry index. **(A)** The orientation asymmetry index was defined as the difference between the left-side normal vector of SC and the mirrored right-side normal vector of SC, the vector reflected in the sagittal plane. **(B)** The position asymmetry index was defined as the distance between a centroid of left-side SC centerlines and a mirrored centroid of right-side SC centerlines. Each side of the centroid corresponded to the mean position of a point cloud of three SC centerlines.

Morphological analysis was performed by one author (T.S.) using ScanIP (Synopsys Inc., Mountain View, USA). In this study, morphological asymmetry in bilateral vestibular organs was quantified using the following procedure (Figure 1):

1. The vestibular organs and eyes were extracted from CISS and T1-weighted images, respectively, through intensity thresholding, and were each reconstructed as a three-dimensional surface shape model.
2. The centerline of each SC was estimated as point cloud data with a sampling interval of 0.04 mm.
3. The normal vector of each SC was determined as the third principal component axis of the centerline point cloud data. The orientations of the normal vectors were determined as posterior to anterior (for anterior and posterior SCs) or inferior to superior (for horizontal SCs).
4. The sagittal plane was defined by regarding a connecting vector between the eyes' centroids as its normal vector and the center of the eyes' centroids as its position (Figure 1B). The centroid of each eye was estimated by least squares fitting of a sphere to the eye's surface shape model after removal of 30% of its anterior part because of the cornea's low sphericity.
5. Vestibular morphological asymmetry was evaluated using two different indices. One was the orientation asymmetry index, defined as the difference between the SC normal vector and the mirrored normal vector, with respect to the sagittal plane, of the contralateral SC (Figure 2A). The other was the position asymmetry index, defined as the distance between the centroid of left SC centerlines and the mirrored centroid of right SC centerlines (Figure 2B). These indices are zero when bilateral vestibular organs are mirror symmetrical with respect to the sagittal plane.

Both orientation and position asymmetry indices were compared between the LS and HS groups using a two-tailed Welch's *t*-test with a significance level of *P* < 0.05.

### 2.4. Functional Connectivity Analysis

For each participant, EPI images were first realigned to correct for head motions using the SPM12 package (v7487; https://www.fil.ion.ucl.ac.uk/spm). Next, the realigned EPI images were spatially corrected using the FSL topup (version 5.0.8; https://fsl.fmrib.ox.ac.uk/fsl/ fslwiki/topup) with a pair of spin-echo EPI images acquired in opposite phase-encoding directions. For the normalization process, a deformation field to MNI space was estimated from a T1-weighted image with diffeomorphic anatomical registration using exponentiated Lie algebra (Ashburner, 2007) implemented in the CAT12 toolbox (r1184; http://www.neuro.uni-jena.de/cat), and this was applied to the corrected EPI images. The normalized EPI images were smoothed using an isotropic Gaussian kernel with a full-width half-maximum of 8 mm.

To identify the impacts of morphological asymmetry in bilateral vestibular organs on cortical vestibular networks, seed-to-voxel resting-state functional connectivity analysis was performed using the CONN toolbox with the default settings (version 17.f; https://www.nitrc.org/forum/projects/conn). In short, the preprocessed EPI images were further processed using the anatomical component correction method (aCompCor) (Behzadi et al., 2007) to regress out physiological noises, and to scrub volumes compromised with motion artifacts using the ART toolbox (https://www.nitrc.org/projects/ artifact_detect). The seed regions were anatomically determined according to a previous study by Indovina et al. (2020), which aimed at identifying the cortical networks of vestibular core regions, namely the posterior insular vestibular cortex (PIVC) and the parietoinsular cortex (PIC): for the left PIVC, *x* = −36, *y* = −25, *z* = +18; for the right PIVC, *x* = +36, *y* = −22, *z* = +17; for the left PIC, *x* = −46, *y* = −33, *z* = +24; for the right PIC, *x* = +51, *y* = −27, and *z* = +28. For each seed, functional connectivity correlated with each morphological asymmetry index was explored. Statistical significance was set at an uncorrected voxel-wise threshold of *P* < 0.001 and a family-wise error (FWE)-corrected cluster-level threshold of *P* < 0.05 for multiple comparisons.

## 3. Results

We first summarized the morphological characteristics of SCs. Each pair of unilateral SC planes are known to be approximately perpendicular (≈90°) (Blanks et al., 1975; Bradshaw et al., 2010). Similarly, the angles between the corresponding bilateral pair of SC planes are approximately perpendicular in anterior and posterior SCs and horizontal in horizontal SC (Bradshaw et al., 2010). To confirm these basic characteristics, we evaluated the angles between any pair of SC normal vectors across all participants and consequently obtained comparable results to those from a previous study using high-resolution computed tomography (Bradshaw et al., 2010) (“all” column in Table 1). We also evaluated the distance from the sagittal plane to a point cloud centroid of SC centerline. Consistent with the results of a previous MRI study (Hitier et al., 2015), the results showed it measured around 40 mm (“all” column in Table 2). Finally, we compared these morphological characteristics (angle and distance) between the LS and HS groups, finding no significant differences between groups (“LS,” “HS,” and “two-tailed *P*” columns in Tables 1, 2).

**Table 1:** Angles between each pair of SCs.

**Angle**	**LS**	**HS**	**All**	**Two-tailed** ***P***
ASC and HSC (left)	91.0 (8.78)	91.0 (7.01)	91.0 (7.89)	0.98
ASC and HSC (right)	89.2 (6.83)	92.0 (6.78)	90.6 (6.90)	0.088
ASC and PSC (left)	94.6 (4.83)	93.9 (4.99)	94.3 (4.89)	0.57
ASC and PSC (right)	92.8 (4.85)	94.3 (4.34)	93.6 (4.63)	0.18
HSC and PSC (left)	85.9 (7.91)	84.8 (5.86)	85.4 (6.94)	0.49
HSC and PSC (right)	86.8 (4.23)	86.8 (5.54)	86.8 (4.90)	0.97
Bilateral ASC	103 (7.42)	102 (9.44)	103 (8.45)	0.53
Bilateral HSC	14.2 (8.74)	12.7 (6.73)	13.5 (7.78)	0.42
Bilateral PSC	83.0 (7.44)	85.5 (7.42)	84.2 (7.49)	0.15

*Values are mean (standard deviation) in degrees. HS, highly susceptible individuals to motion sickness; LS, age/sex-matched low susceptibility controls; ASC, anterior SC; HSC, horizontal SC; PSC, posterior SC*.

**Table 2:** Distance between sagittal plane and each SC pair.

**Distance**	**LS**	**HS**	**All**	**Two-tailed** ***P***
Sagittal-ASC (left)	40.5 (1.83)	40.2 (1.92)	40.4 (1.87)	0.45
Sagittal-ASC (right)	40.7 (1.79)	40.3 (1.90)	40.5 (1.84)	0.34
Sagittal-HSC (left)	42.9 (1.85)	42.6 (1.94)	42.8 (1.89)	0.44
Sagittal-HSC (right)	42.8 (1.84)	42.5 (1.97)	42.7 (1.90)	0.47
Sagittal-PSC (left)	40.8 (1.82)	40.4 (1.99)	40.6 (1.90)	0.49
Sagittal-PSC (right)	40.7 (1.86)	40.4 (2.06)	40.6 (1.95)	0.50

*Values are mean (standard deviation) in mm*.

**Figure 3:**
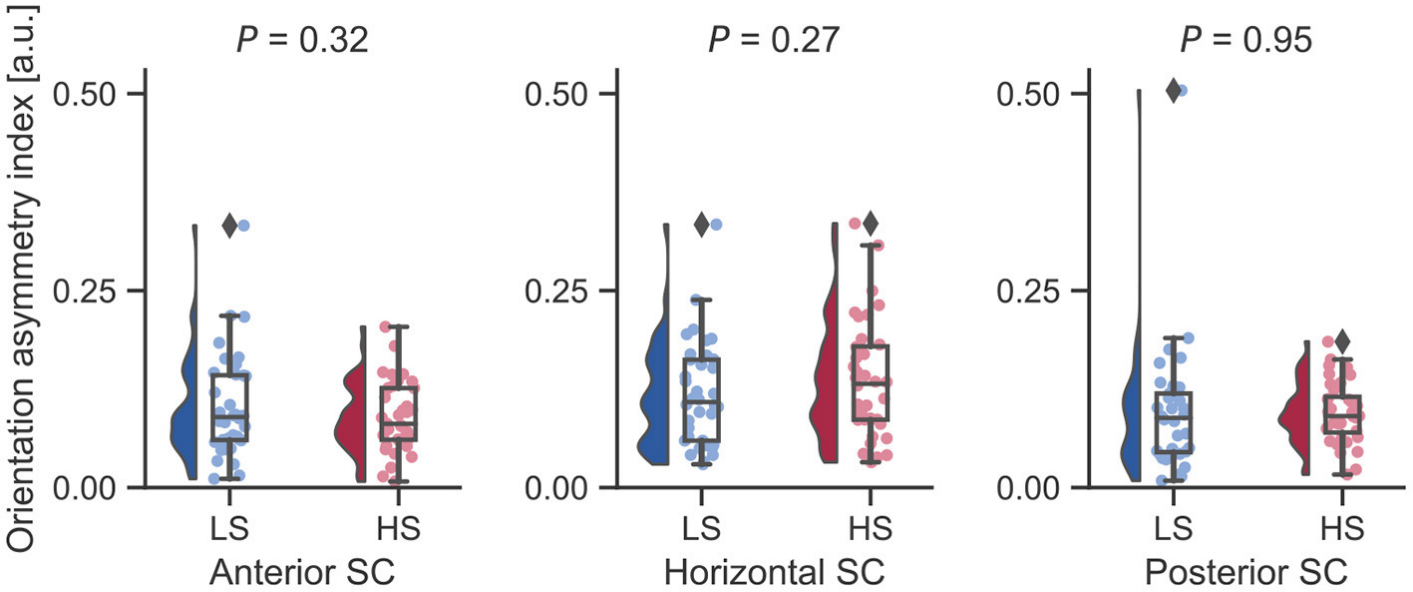
Between-group comparisons of orientation asymmetry index for each bilateral SC pair. There were no significant differences on a two-tailed Welch's *t*-test. Diamonds represent outliers.

**Figure 4:**
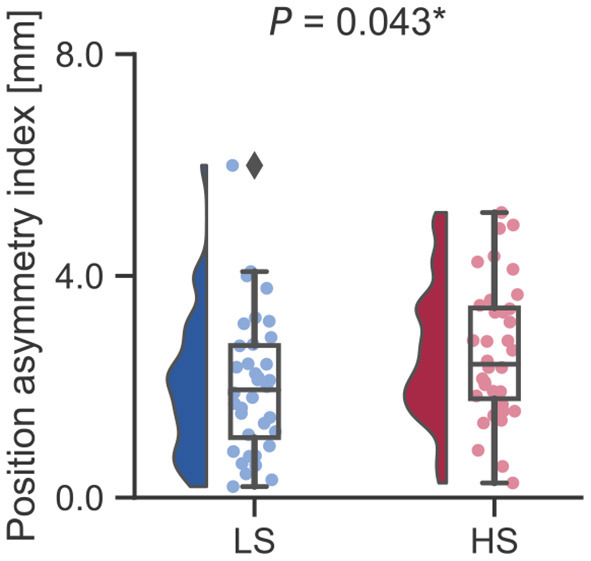
Between-group comparison of the position asymmetry index. The position asymmetry index was significantly higher in the HS group than in the LS group (LS: mean, 2.0; 95% CI, 1.6 to 2.4; HS: mean, 2.6; 95% CI, 2.2 to 3.0; two-tailed Welch's *t*-test, *P* = 0.043). ^*^P < 0.05. A Diamond represents an outlier.

Next, we examined whether vestibular morphological asymmetry was associated with motion sickness susceptibility. No significant between-group differences in the orientation asymmetry index for each SC were found (Figure 3). To more closely evaluate the orientation asymmetry index, we further compared it in each axis. There were no significant differences between groups in any axis (Table 3). In contrast, there was a significant between-group difference in the position asymmetry index [LS: mean, 2.0; 95% confidence interval (CI), 1.6 to 2.4; HS: mean, 2.6; 95% CI, 2.2 to 3.0; two-tailed Welch's *t*-test, *P* = 0.043; Figure 4]. Closer evaluation revealed no significant between-group difference in the position asymmetry index for each axis (Table 4), indicating that the position asymmetry associated with motion sickness susceptibility was not axis-specific.

**Table 3:** Orientation asymmetry index in each axis.

**SC**	**Axis**	**LS**	**HS**	**All**	**Two-tailed** ***P***
Anterior SC	X	0.035 (0.030)	0.040 (0.035)	0.038 (0.033)	0.53
	Y	0.039 (0.036)	0.035 (0.028)	0.037 (0.032)	0.56
	Z	0.079 (0.061)	0.060 (0.041)	0.069 (0.052)	0.12
Horizontal SC	X	0.069 (0.058)	0.087 (0.058)	0.078 (0.058)	0.17
	Y	0.085 (0.054)	0.090 (0.071)	0.087 (0.063)	0.75
	Z	0.013 (0.012)	0.013 (0.0099)	0.013 (0.011)	0.81
Posterior SC	X	0.031 (0.027)	0.034 (0.029)	0.033 (0.028)	0.71
	Y	0.034 (0.024)	0.034 (0.031)	0.034 (0.028)	0.93
	Z	0.075 (0.085)	0.073 (0.035)	0.074 (0.065)	0.87

*Values are mean (standard deviation) in arbitrary units*.

**Table 4:** Position asymmetry index in each axis.

**Axis**	**LS**	**HS**	**All**	**Two-tailed** ***P***
X	1.1 (0.93)	1.4 (1.3)	1.3 (1.1)	0.24
Y	0.044 (0.053)	0.048 (0.040)	0.046 (0.046)	0.69
Z	1.4 (1.2)	1.9 (1.1)	1.7 (1.2)	0.099

*Values are mean (standard deviation) in mm*.

**Figure 5:**
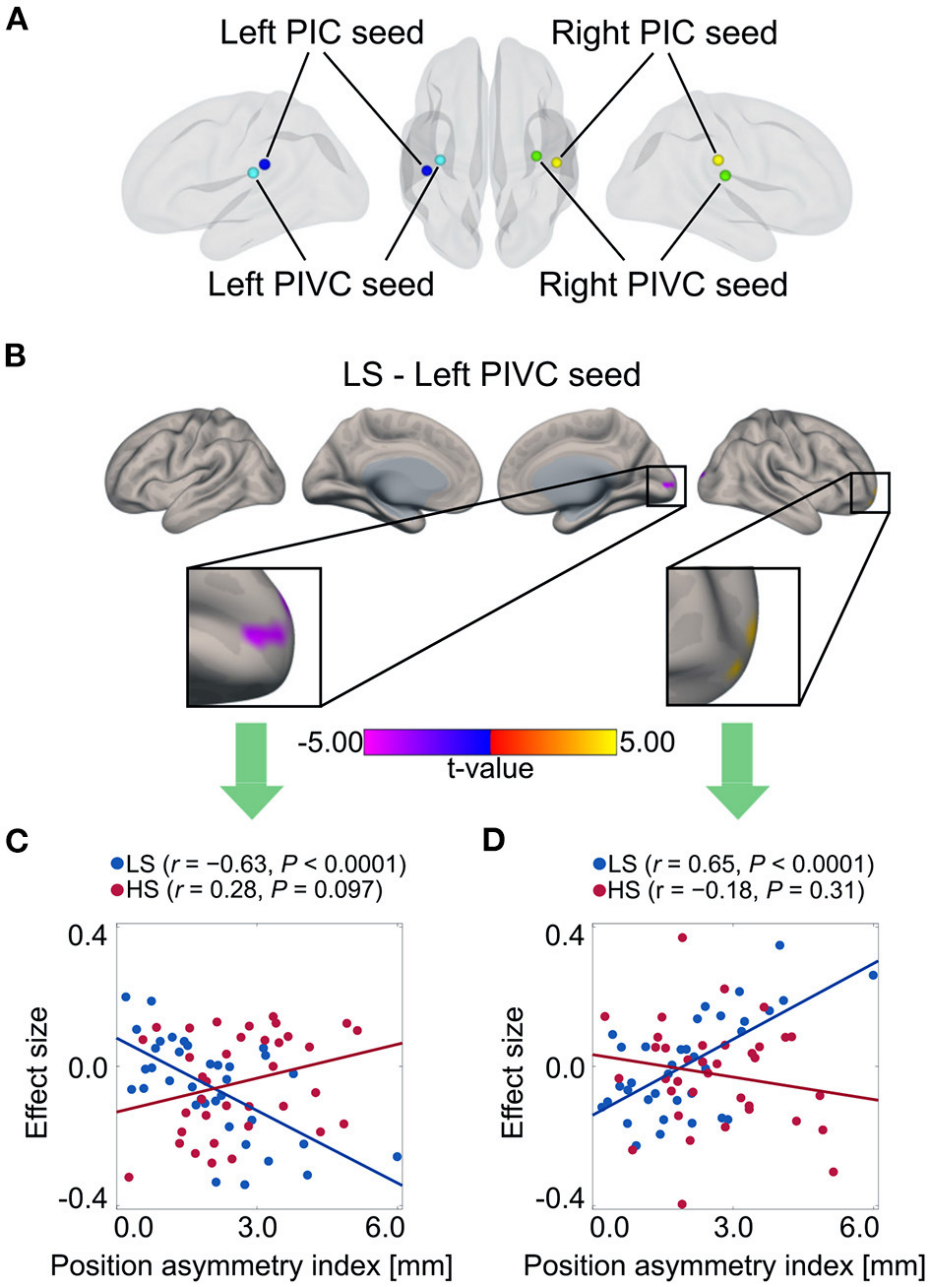
Correlation between position asymmetry index and functional connectivity of cortical vestibular networks in each group. **(A)** Vestibular seed regions used in seed-based resting-state functional connectivity analysis. Correlations in left PIVC seeds were found only in LS group. **(B)** Negative correlation between position asymmetry index and functional connectivity between left PIVC and medial occipital cortex in the LS group. Positive correlation between position asymmetry index and functional connectivity between left PIVC and right lateral frontal pole in the LS group. **(C)** Scatter plot of position asymmetry index and functional connectivity between the left PIVC and medial occipital cortex in each group. There was a negative correlation in the LS group, but not in the HS group. **(D)** Scatter plot of position asymmetry index and functional connectivity between the left PIVC and the right lateral frontal pole in each group. There was a positive correlation in the LS group, but not in the HS group. PIC, parietoinsular cortex.

Finally, to explore the impacts of vestibular morphological asymmetry on cortical vestibular networks, we performed a regression analysis of functional connectivity in each group, using the position asymmetry index as covariate. In the LS, but not the HS group, there was a correlations between the position asymmetry index and functional connectivity in the cortical vestibular network. Specifically, the position asymmetry index was negatively correlated with functional connectivity between the left PIVC seed (Figure 5A) and the medial occipital cortex (cluster-level FWE-corrected *P* = 0.013, Figure 5B and Table 5). The mean functional connectivity within this cluster again confirmed that a negative correlation existed only in the LS but not the HS group (LS: *r* = −0.63, *P* < 0.0001; HS: *r* = 0.28, *P* = 0.097; Figure 5C). In addition, we found a positive correlation between the position asymmetry index and the functional connectivity between the left PIVC seed and the right frontal pole (cluster-level FWE-corrected *P* = 0.046, Figure 5B and Table 5; LS: *r* = 0.65, *P* < 0.0001; HS: *r* = −0.18, *P* = 0.31, Figure 5D).

**Table 5:** Correlation between position asymmetry index and functional connectivity in LS group.

**Seed**	**MNI coordinates** **(x, y, z)**	**Brain region** **(AAL)**	**Cluster size**	**cluster level** ***P*** **(FWE-corrected)**	**Correlation**
left PIVC	+18, −96, +10	Cuneus R	130	0.013	LS: −0.63 (*P* <0.0001)
					[HS: 0.28 (*P* = 0.097)]
	+26, +66, −14	Frontal Mid Orb R*^a^*	99	0.046	LS: 0.65 (*P* <0.0001)
					[HS: −0.18 (*P* = 0.31)]

*AAL, automated anatomical labeling; PIVC, posterior insular vestibular cortex*.

a *nearest label*.

## 4. Discussion

Motion sickness is a long-standing issue associated with human mobility currently under the spotlight because of the rise of autonomous vehicle technologies. In the current study, using inner ear MRI, we examined whether vestibular morphological asymmetry is associated with motion sickness susceptibility. As a result, we found a larger vestibular position asymmetry in individuals with high rather than low susceptibility to motion sickness. In addition, vestibular position asymmetry correlated with resting state functional connectivity between vestibular and visual cortical regions in lowly, but not highly, susceptible individuals.

A key finding in this study is that vestibular morphological asymmetry is associated with motion sickness susceptibility. In previous studies, patients with scoliosis were found to show higher orientation asymmetry in bilateral horizontal SCs (Hitier et al., 2015; Carry et al., 2020) and higher susceptibility to motion sickness than healthy controls (Catanzariti et al., 2016). From these findings, we expected that the orientation asymmetry index would be associated with motion sickness susceptibility. Contrary to our expectation, however, the current results showed that the position asymmetry index, which was not evaluated in those previous studies, is associated with motion sickness susceptibility in healthy individuals.

**Figure 6:**
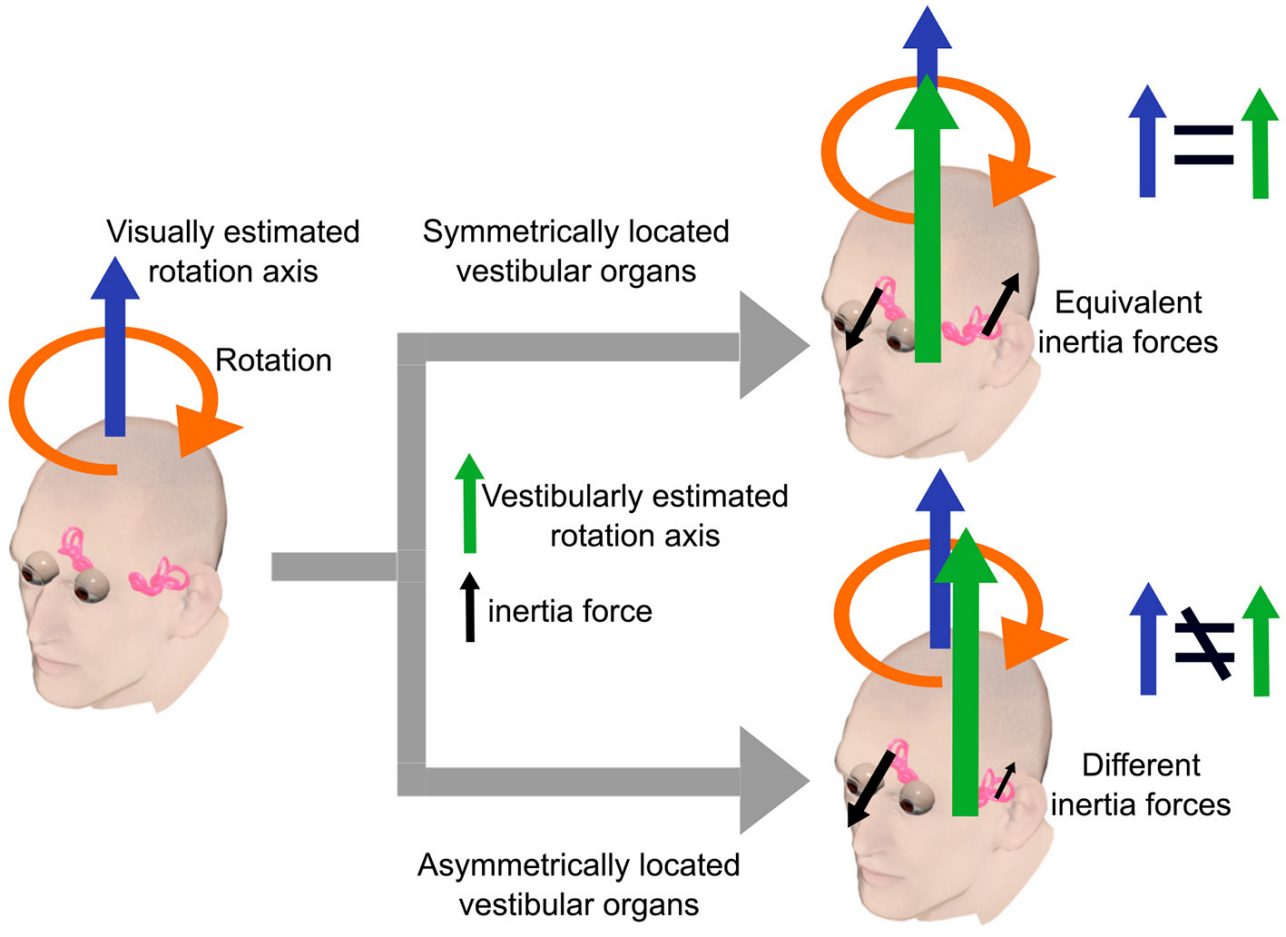
A simple example illustrating our hypothesis. The head rotates around an axis within the sagittal plane equidistant from both eyes. In this case, the visually estimated rotation axis lies in the sagittal plane. The vestibularly estimated rotation axis lies in the sagittal plane when the bilateral vestibular organs are symmetrically located with respect to the sagittal plane. In contrast, vestibularly estimated axis lies outside the sagittal plane when the vestibular organs are asymmetrically located due to unbalanced centrifugal forces on the bilateral vestibular organs.

An emerging question is the type of sensory conflicts induced by the vestibular morphological asymmetry and associated with motion sickness susceptibility. A likely possibility is the dissociation of rotation axes estimated from visual and vestibular information, respectively. For simplicity, suppose that the head rotates around an axis within the sagittal plane equidistant from both eyes (see also Figure 1B). In this case, while the visually estimated rotation axis would lie in the sagittal plane, the vestibularly-estimated one would be out of the sagittal plane because of unbalanced centrifugal forces on the bilateral vestibular organs (Figure 6). This dissociation can result in sensory conflicts in terms of head rotation and, consequently, motion sickness. Supporting this notion, scoliosis patients, known to show vestibular morphological asymmetry (Hitier et al., 2015; Carry et al., 2020), show alterations in the perception of rotation (Simoneau et al., 2009) and verticality (Cakrt et al., 2011), which require integration of vestibular signals. This notion implicates that motion sickness results from multi-/intersensory (e.g., vision and vestibular senses) conflicts due to intrasensory inconsistencies, thus extending the sensory conflict theory.

Furthermore, our resting-state functional MRI analysis revealed that vestibular morphological asymmetry was associated with cortical vestibular network alterations. Specifically, the position asymmetry index was negatively correlated with functional connectivity between the left PIVC and the occipital visual cortex only in the LS group. According to previous neuroimaging studies, visual-vestibular reciprocal inhibitory interactions (Brandt and Dieterich, 1999), namely, activation in vestibular cortical areas and deactivation in visual cortical areas and vice versa, is hypothesized to play a role in resolving sensory conflicts between the visual and vestibular senses by reducing the weight of less reliable sensory signals (Brandt et al., 1998; Dieterich and Brandt, 2015). Despite the controversial issues on the interpretation of negative functional connectivity (Goelman et al., 2014), our results are consistent with this hypothesis of visual-vestibular reciprocal inhibitory interactions. In addition, in individuals whose resolving mechanism does not work properly, sensory conflicts due to vestibular morphological asymmetry tend to appear, resulting in motion sickness susceptibility.

Our results also showed that the position asymmetry of vestibular organs correlated with a vestibular network connected with the frontal pole. Although few studies have reported on this region in relation to motion sickness, a previous structural study may provide further insights into motion sickness mechanisms. For example, the frontal pole is connected to high-order sensory and visual cortices and considered to play a role in attention and behavior control (Orr et al., 2015), even though the frontal pole function in humans mostly remains unclear. This finding, together with our results, may imply that functions guiding behavior also characterize individual motion sickness susceptibility, but further research is warranted.

The present study has some limitations that should be addressed in future research. First, we may have overlooked some group differences due to the lower prediction reliability of MSSQ score for motion sickness insusceptibility. As noted in the MSSQ (Golding, 2006) and recent literature (Leilei et al., 2021), the MSSQ score in the “low susceptible” zone shows greater deviation in the correlation analysis between MSSQ score and nausea latency. Furthermore, the low MSSQ score group includes participants with low to middle susceptibility, as reported by Golding (2006). Accordingly, this may lead to low statistical power that increases the probability of type II errors and decreases the probability of detecting group differences. We note that identifying individuals resistant to motion sickness is difficult. As discussed in the MSSQ paper (Golding, 2006), laboratory motion stimuli are not strongly correlated with self-reported susceptibility (Reason and Brand, 1975; Golding, 1989), and some studies have implicated individual differences in motion sensitivity to different motion types (Lentz, 1984; Golding, 1993). Given that a combination of many motion types can cause motion sickness in real-life situations, the prediction of motion sickness susceptibility from laboratory experiments has some limitations. Hence, for stricter investigation of motion sickness resistance, a screening test in real-life situations for grouping participants is warranted. Second, there is some interindividual variability of vestibular areas, even though we used vestibular seeds based on a previous study that reported structural differences in connectivity patterns between the PIVC and PIC (Indovina et al., 2020). Particularly, for PIVC and PIC localization, the vestibular areas should be identified by visual and vestibular localization experiments and defined in a subject-specific manner. Third, we assessed visual-vestibular reciprocal interactions using resting-state functional MRI. In the future, the degree of negative functional connectivity and the stimulus-evoked activation/deactivation balance between visual and vestibular regions should be directly compared. Fourth, we only focused on morphological asymmetry of the bilateral SCs. Another insight into motion sickness pathogenesis may be identified by examining the impacts of geometrical misalignment in bilateral otoliths on motion sickness susceptibility since studies on sea sickness have demonstrated that linear acceleration can provoke motion sickness (Bertolini and Straumann, 2016). Further investigation using a specialized MRI sequence to analyze the position and posture of the otolith (Naganawa and Nakashima, 2014; Thylur et al., 2017) is warranted to confirm this hypothesis. Fifth, our study did not show any direct evidence of the functional impacts of morphological vestibular asymmetry on spatial orientation, despite the existence of a central compensation of unbalanced vestibular afferent signals (Lacour et al., 2016).

## 5. Conclusion

Our results provide the first evidence that the morphological asymmetry of vestibular organs is a determinant factor of motion sickness susceptibility in healthy individuals and closely associated with the functional vestibular network, particularly relating to visual-vestibular reciprocal interactions. These findings thus advance the understanding of motion sickness pathogenesis and provide neural insight underlying visual-vestibular weights. Further understanding of visual-vestibular weighting may also help design novel visual auxiliary systems for vehicles and transportation systems that assist weighting vision thereby preventing motion sickness, leading to better mobility from a neuroergonomic perspective.

## Data Availability Statement

The raw data supporting the conclusions of this article will be made available by the authors, without undue reservation.

## Ethics Statement

The studies involving human participants were reviewed and approved by the Ethics Committees of both Toyota Central R&D Labs., Inc., and the National Institute for Physiological Sciences. The patients/participants provided their written informed consent to participate in this study.

## Author Contributions

TH analyzed the data and wrote the manuscript. TS conducted the experiments and analyzed the data. SL and AD supervised the functional analysis. TI and YW conducted the medical diagnoses. MF and NS acquired the MRI data. SL conceived the functional analysis idea. HS designed the experiments, revised the manuscript, and supervised the project. All authors provided feedback on the manuscript.

## Conflict of Interest

TH, TS, and HS were employed by Toyota Central R&D Labs. The remaining authors declare that the research was conducted in the absence of any commercial or financial relationships that could be construed as a potential conflict of interest.

## Publisher's Note

All claims expressed in this article are solely those of the authors and do not necessarily represent those of their affiliated organizations, or those of the publisher, the editors and the reviewers. Any product that may be evaluated in this article, or claim that may be made by its manufacturer, is not guaranteed or endorsed by the publisher.
